# Pulse of Children at the Breast

**Published:** 1842-04

**Authors:** 


*Pulse of Children at the Breast.*—M. TROUSSEAU has inserted in the *Journ. des Connaissances, Med. Chirurg.* an interesting memoir on this subject. He gives first an abstract of what MM. Billard, Valleix, and others, have written on the pulse of infants at the breast. From them we find that before birth, and during pregnancy, the average of the pulse is 133 in the minute; that it falls to 83 at the moment the child is expelled; and that in some minutes after it rises to 160. In the course of the first day it falls again to 127, and continues gradually to diminish during the ten first days, its average being then from 87 to 90 per minute. The above numbers are only the average formed, as the variations of the pulse are very great in the new born infant. The following are the results which M. Trosseau has obtained. In infants from eight days to six months old, the average number of pulsations for boys was 131, and for girls 134; from six to twenty-one months, the average for boys was 113, and for girls 126, but along with the authors above cited, he found that the extremes were frequently far above or below the average.

The states of waking and sleeping have a much more sensible influence on the pulse than sex. M. Trousseau found that in infants from fifteen days to six months old, the average of the pulse was 140 during waking, and 121 during sleep; and in infants from six to twenty-one months, 128 during waking, and 112 during sleep; and this difference is still more marked when the child is afraid, cries, or struggles, when the physician is feeling its pulse. In these cases he has seen it rise from 112 to 160 and 180.

To sum up—the pulse of children at the breast varies from 100 to 150. After the first two months, it is a little more frequent in females than in males; and it is about twenty pulsations higher in a state of waking than it is during sleep.

The most important result of these researches is to show the impossibility of discovering a febrile state in infants, from the pulse alone.—*Am. Journ. Med. Sciences* from *Journ. de Med. et de Chirurg. Prat.*, Aug. 1841.

